# Developing a Technology Acceptability and Usage Survey (TAUS) for mHealth Intervention Planning and Evaluation in Nigeria: Pilot Study

**DOI:** 10.2196/34035

**Published:** 2022-04-20

**Authors:** Kathleen A Lynch, Thomas M Atkinson, Adeleye D Omisore, Olusola Famurewa, Olalekan Olasehinde, Oluwole Odujoko, Olusegun I Alatise, Adedeji Egberongbe, T Peter Kingham, Elizabeth A Morris, Elizabeth Sutton

**Affiliations:** 1 Department of Psychiatry & Behavioral Sciences Memorial Sloan Kettering Cancer Center New York, NY United States; 2 Department of Radiology Obafemi Awolowo University Ile-Ife Nigeria; 3 Department of Surgery Obafemi Awolowo University Ile-Ife Nigeria; 4 Department of Pathology Obafemi Awolowo University Ile-Ife Nigeria; 5 Department of Radiology Federal Medical Centre Owo Nigeria; 6 Department of Surgery Memorial Sloan Kettering Cancer Center New York, NY United States; 7 Department of Radiology Memorial Sloan Kettering Cancer Center New York, NY United States

**Keywords:** measure development, survey methods, technology acceptability and use, global health, mHealth, Nigeria

## Abstract

**Background:**

Technology acceptability and usage surveys (TAUS) are brief questionnaires that measure technology comfort, typical daily use, and access in a population. However, current measures are not adapted to low- and middle-income country (LMIC) contexts.

**Objective:**

The objective of this pilot study was to develop a TAUS that could be used to inform the implementation of a mobile health (mHealth) intervention in Nigeria.

**Methods:**

A literature review of validated technology comfort and usage scales was conducted to identify candidate items. The draft measure was reviewed for face validity by an expert panel comprised of clinicians and researchers with cultural, methodological, and clinical expertise. The measure was piloted by radiologists at an oncology symposium in Nigeria.

**Results:**

After expert review, the final measure included 18 items organized into 3 domains: (1) comfort with using mobile applications, (2) reliability of internet or electricity, and (3) attitudes toward using computers or mobile applications in clinical practice. The pilot sample (n=16) reported high levels of comfort and acceptability toward using mHealth applications in the clinical setting but faced numerous infrastructure challenges.

**Conclusions:**

Pilot results indicate that the TAUS may be a feasible and appropriate measure for assessing technology usage and acceptability in LMIC clinical contexts. Dedicating a domain to technology infrastructure and access yielded valuable insights for program implementation.

## Introduction

### Background

Over the past decade, mobile Health (mHealth)–based interventions have become affordable, accessible options for health education and care, particularly in low- and middle-income country (LMIC) contexts [1,2]. Developing an mHealth-based intervention typically involves iterative cycles of design, testing, and rapid adaptation, as well as retesting in the targeted population. Technology acceptability and usage surveys (TAUS) may streamline these cycles by providing insight into the social context of device implementation and use. TAUS are brief questionnaires that measure technology comfort, typical daily use, and access in a population. Although TAUS have been used to inform digital interventions such as telehealth [3], they are seldom adapted to LMIC contexts or developed in the local language, despite their potential to support pre-implementation planning.

For global health intervention planning, there is a need for validated TAUS that are appropriate for use in LMIC settings, particularly in locations with limited electricity or digital infrastructure. Previously developed TAUS are either out of date, referencing technology platforms that no longer exist (eg, Alta Vista), or were developed in settings with large technology infrastructures such as the United States [4]. Additionally, previous literature also indicates that the use of mobile devices is culturally patterned [5,6]. For instance, in sub-Saharan Africa, the use of mobile applications has “leapfrogged” over computer-based platforms as a primary tool for work information-seeking in digital spaces [7]. Thus, it is necessary to develop TAUS tools that are not only adapted to local infrastructure but also tailored to the local cultural context.

### Goal of This Study

The objective of this pilot study was to develop and pilot a TAUS that could be used to inform the implementation of an mHealth intervention in Nigeria. In this paper, we describe our methods for developing the TAUS and preliminary insights from piloting the measure among a group of Nigerian health professionals.

### Setting

Nigeria is the most populous country in Africa and has the highest breast cancer mortality rate [8]; breast cancer is the leading cause of death among Nigerian women [9]. Although breast cancer is typically diagnosed via ultrasound-guided breast biopsy in most high-income countries, this approach is less frequently available in LMICs due to the prohibitive costs of imaging devices, materials, and maintenance [10]. In the absence of imaging, breast cancer is commonly diagnosed in LMICs either through blind biopsy, which is less accurate, or surgical excision, which increases morbidity [10].

However, battery-operated, mHealth tablet-based devices have the potential to expand ultrasound access in LMICs. An mHealth tablet-based ultrasound device is both less expensive and less costly than hospital-grade ultrasound machines. This device consists of a portable, high-frequency, ultrasound probe that attaches by USB to either a tablet or smartphone, which displays and send images to a secure mobile application. The device proposed for use in this intervention was developed in the United States (Philips Ultrasound Inc, Bothell, WA) but has been successfully used by midwives at Aga Khan hospital in Nairobi, Kenya [11]. However, this device has not been piloted in oncology settings nor within the context of the Nigerian hospital system. Therefore, this project presented an ideal opportunity to develop and administer a novel TAUS to help us understand radiologists’ current technology use, access, and comfort in clinical settings, which would inform the implementation strategy of this tablet-based ultrasound intervention.

## Methods

### Measure Development

To select candidate items for the measure, we conducted a literature search of questionnaires related to physician technology usage as well as validated technology comfort and usage scales developed for clinical settings [2,3,12-14]. Drawing on themes identified in the literature, we created a first draft of the TAUS, organized into 4 domains: (1) comfort with using mobile applications; (2) reliability of internet, Wi-Fi, and electricity; (3) utilization of computers or mobile applications in clinical practice; and (4) attitudes toward using computers or mobile applications in clinical practice. Each domain contained 7 to 10 closed-ended or 5-point Likert scale items. The draft measure was then reviewed for face validity by an expert panel comprised of clinicians and researchers with cultural, methodological, and clinical expertise. This review enabled us to tailor the measure to the local Nigerian context and reduce the number of items needed to assess each construct to reduce respondent fatigue. Experts gave feedback independently and then convened to reach consensus on item consolidation and removal during a 60-minute feedback session. After revising the measure, experts re-reviewed and approved the final version prior to piloting.

### Ethical Review

To pilot the measure, we obtained approval from the Institutional Review Board at Memorial Sloan Kettering Cancer Center (number IRB 18-114) for our study. All pilot study procedures were performed in accordance with the 1964 Helsinki declaration and its later amendments. Informed consent was obtained from all individual participants included in the study.

### Piloting the Measure

Data collection occurred in April 2019 at the sixth annual symposium of the African Research Group for Oncology (ARGO), a National Cancer Institute–recognized cancer consortium that aims to improve outcomes for cancer patients in Nigeria. The symposium was hosted by Obafemi Awolowo University Teaching Hospital in Ile-Ife, Nigeria. This setting was chosen because it presented an opportunity to simultaneously survey Nigerian radiologists working in diverse community and geographic contexts. During the conference, radiologist attendees attended a didactic and training session focused on mHealth tablet-based ultrasound device–guided breast biopsy. Following a hands-on demonstration of the mHealth device, radiologists completed a paper copy of the TAUS. Responses were analyzed descriptively.

## Results

After expert review of the responses, we consolidated “Comfort” and “Utilization” into a single domain given the overlap in constructs, expanded the “Reliability” domain to 10 items to reflect local infrastructure challenges, and reduced the “Attitudes” domain to 4 key items to reduce potential respondent fatigue. The final measure included 18 items organized into 3 domains: (1) comfort with using mobile applications (n=4), (2) reliability of internet and electricity (n=10), and (3) attitudes toward using computers or mobile applications in clinical practice (n= 4). The full measure can be found in Multimedia Appendix 1.

The survey was completed by 16 radiologists (Table 1). The survey took respondents approximately 5 minutes to complete. Respondents reported high levels of comfort and acceptability toward using mHealth applications in clinical settings. However, they reported a low level of technology infrastructure: 12 (12/16, 75%) reported that their hospital does not use an electronic medical record, and 9 (9/16, 56%) indicated that they are responsible for funding their own internet/Wi-Fi at their clinical practice. Approximately two-thirds (10/16, 63%) indicated that their hospital loses electricity more than once per day; 13 (13/16, 81%) noted that when electricity goes out, internet access is also disabled. In addition, 9 (9/16, 56%) indicated that it can take over 1 month for a malfunctioning device to be repaired at their hospital.

**Table 1:** Results of the technology comfort and use survey (n=16) in Ile-Ife, Nigeria in 2019.

Questions and Responses			Results, n (%)
**Comfort with using mobile applications: “How comfortable are you with using computers or mobile applications for clinical purposes?”**			
	Very comfortable	8 (50)	
	Extremely comfortable	8 (50)	
**Comfort with using mobile applications: “How comfortable are you with using computers or mobile applications for professional educational purposes?”**			
	Somewhat comfortable	1 (6)	
	Very comfortable	7 (44)	
	Extremely comfortable	8 (50)	
**Utilization of computers or mobile applications in clinical practice: “I have taken an online/e-learning course in the past.”**			
	No	3 (19)	
	Yes	13 (81)	
**Utilization of computers or mobile applications in clinical practice: “What is the primary way you communicate clinical information with your colleagues?”**			
	In-person	1 (6)	
	Phone call	6 (38)	
	Multiple forums (including text message, mobile application)	9 (56)	
**Technology access: Reliability of internet, Wi-Fi, and electricity: “I have reliable access to the internet/Wi-Fi at my hospital.”**			
	Disagree	2 (13)	
	Neutral	1 (6)	
	Agree	9 (56)	
	Strongly agree	3 (19)	
**Technology access: Reliability of internet, Wi-Fi, and electricity: “Who provides funding for your access to the internet/Wi-Fi for your clinical practice?”**			
	Self	9 (56)	
	Hospital	1 (6)	
	Combination of self and hospital	6 (38)	
**Technology access: Reliability of internet, Wi-Fi, and electricity: “What proportion of your average day do you have access to internet/Wi-Fi for your clinical practice?”**			
	Entire day	5 (31)	
	Most of the day	7 (44)	
	Half of the day	2 (13)	
	Less than half of the day	2 (13)	
**Technology access: Reliability of internet, Wi-Fi, and electricity: “What is the connection type for intranet/internet/Wi-Fi that you use for your clinical practice?”**			
	Dial-up	1 (6)	
	Wireless broadband	15 (94)	
**Technology access: Reliability of internet, Wi-Fi, and electricity: The hospital loses electricity.**			
	1 per day	2 (13)	
	>1 per day	10 (63)	
	1-3 times per week	4 (25)	
**Technology access: Reliability of internet, Wi-Fi, and electricity: “Does your hospital have a generator?”**			
	No	0 (0)	
	Yes	15 (94)	
	Missing	1 (6)	
**Technology access: Reliability of internet, Wi-Fi, and electricity: “When the electricity goes out, how long does it take to come back on?”**			
	<1 hour	6 (38)	
	1-2 hours	4 (25)	
	2-3 hours	3 (19)	
	4-6 hours	1 (6)	
	>12 hours	1 (6)	
	Missing	1 (6)	
**Technology access: Reliability of internet, Wi-Fi, and electricity: “When the electricity goes out at your hospital does this also disable the internet/ Wi-Fi access?”**			
	No	2 (13)	
	Yes	13 (81)	
	My hospital does not have internet/Wi-Fi	1 (6)	
**Technology access: Reliability of internet, Wi-Fi, and electricity: “When a device malfunctions at your hospital how long does it take to get a repair?”**			
	<7 days	2 (13)	
	7-14 days	2 (13)	
	15-30 days	3 (19)	
	≥31 days	9 (56)	
**Technology access: Reliability of internet, Wi-Fi, and electricity: “Does your hospital have an electronic medical record?”**			
	No	12 (75)	
	Yes	3 (19)	
	Unsure	1 (6)	
**Attitudes toward using computers or mobile applications in clinical practice**: **“I find online/e-learning courses to be helpful.”**			
	Strongly disagree	1 (6)	
	Neutral	1 (6)	
	Agree	3 (19)	
	Strongly agree	9 (56)	
**Attitudes toward using computers or mobile applications in clinical practice: “Using a computer or mobile application makes it easier to maintain or improve the health condition of my patients.”**			
	Agree	4 (25)	
	Strongly agree	12 (75)	
**Attitudes toward using computers or mobile applications in clinical practice: “I think that using a computer or mobile health application to assist with maintaining or improving the health condition of my patients fits well within my clinical practice.”**			
	Agree	4 (25)	
	Strongly agree	12 (75)	
**Attitudes toward using computers or mobile applications in clinical practice: “It is easy for me to use a computer or mobile application to assist with maintaining or improving the health condition of my patients.”**			
	Agree	4 (25)	
	Strongly agree	12 (75)	

## Discussion

This pilot study demonstrates promise for TAUS’ applicability and use in LMIC contexts. By including a domain with detailed questions related to technology infrastructure and access, this survey uncovered key concerns for mHealth use, despite high reported levels of technology comfort and acceptance. Although mHealth-based interventions are widely used, it is necessary to evaluate their feasibility and acceptability with TAUS that are tailored to the cultural context of the targeted population. Pilot results indicate that the TAUS is a feasible and appropriate measure for assessing technology usage and acceptability in LMIC clinical contexts. Dedicating a specific domain to technology infrastructure and access yielded valuable insights for program implementation. For example, administering the TAUS in the context of the current project identified the need for precharged or battery-operated devices, given electricity instability. A limitation of this study is the fact that, while participants completed the measure, they did not provide direct feedback on the measure itself. Future directions include cognitive interviews to assess content validity among the target population.

The findings of this study justify the need to create a flexible TAUS that can be tailored to specific settings. The pilot reveals that the TAUS is a promising measure to support mHealth intervention planning in LMIC contexts. Future directions include larger-scale trials to support validation of the TAUS measure.

## Appendix

Multimedia Appendix 1 The Technology Acceptability and Usage Survey (TAUS) Scale.

## Abbreviations

ARGO: African Research Group for Oncology
LMIC: low- and middle-income country
mHealth: mobile health
TAUS: technology acceptability and usage surveys
