# Supplementary material for: Developing a Technology Acceptability and Usage Survey (TAUS) for mHealth Intervention Planning and Evaluation in Nigeria: Pilot Study
Source: JMIR Form Res. 2022 Apr 20;6(4):e34035. doi: 10.2196/34035 (PMC9069275; doi:10.2196/34035)
Supplement: Multimedia Appendix 1 [file formative_v6i4e34035_app1.docx]

**Appendix 1. The TAUS Scale**

**For each of the following statements, select the most appropriate response:**

1. How comfortable are you with using computers or mobile applications for clinical purposes?

| 1 | 2 | 3 | 4 | 5 |
| --- | --- | --- | --- | --- |
| Not at all comfortable | Slightly comfortable | Somewhat comfortable | Very comfortable | Extremely comfortable |

1. How comfortable are you with using computers or mobile applications for professional educational purposes (e.g., online continuing education course, virtual conference attendance)?

| 1 | 2 | 3 | 4 | 5 |
| --- | --- | --- | --- | --- |
| Not at all comfortable | Slightly comfortable | Somewhat comfortable | Very comfortable | Extremely comfortable |

1. I have taken an online/e-learning course in the past (e.g., continuing education)
   - Yes
   - No (Please skip to question 6)
2. What is the primary way you communicate clinical information with your colleagues?
   - In person
   - Phone call
   - Text Message
   - Electronic Medical Record
   - Mobile Application

**For each of the following questions regarding Internet/WIFI access, please provide the best response:**

1. I have reliable access to the internet/WIFI at my hospital

| 1 | 2 | 3 | 4 | 5 |
| --- | --- | --- | --- | --- |
| Strongly disagree | Disagree | Neutral | Agree | Strongly Agree |

1. For what proportion of your average day do you have access to the internet/WIFI for your clinical practice?
   - Entire day
   - Most of the day
   - Half of the day
   - Less than half of the day
   - No internet/WIFI access
2. Who provides funding for your access to the internet/WIFI for your clinical practice?

_______________________________________________

1. What is the connection type for the intranet/internet/WIFI that you use for your clinical practice?
   - Dial-Up
   - Wired Broadband
   - Wireless Broadband
   - Do not know
2. The hospital loses electricity:
   - More than once a day
   - Once a day
   - 1-3 times per wekk
   - 1-3 times per month
   - Never
3. When the electricity goes out, how long does it take to come back on (on average)?

_____________________ hours

1. When the electricity goes out at your hospital, does this also disable the internet/WIFI access?
   - Yes
   - No
   - Electricity does not go out at my hospital
   - My hospital does not have internet/WIFI access
2. When a device malfunctions at your hospital, how long does it take to get a repair?
   - Less than 7 days
   - 8-14 days
   - 15-30 days
   - 31 days or longer
3. Does your hospital use an electronic medical record?
   - Yes
   - No
   - I’m not sure
4. If YES, How do you use the EMR? [Please check all that apply]:
   - Electronically document encounters (e.g., clinic notes)
   - Electronic patient assessment (e.g., patient-reported symptom surveys)
   - Electronically order laboratory test
   - Electronically prescribe medications
   - Electronically communicate with patients/ access online patient educational materials
   - Electronically communicate with clinicians

**For each of the following statements, please indicate your level of agreement:**

1. I find online/e-learning courses to be helpful

| 1 | 2 | 3 | 4 | 5 |
| --- | --- | --- | --- | --- |
| Strongly disagree | Disagree | Neutral | Agree | Strongly Agree |

1. Using a computer or mobile application makes it easier to maintain or improve the health condition of my patients.

| 1 | 2 | 3 | 4 | 5 |
| --- | --- | --- | --- | --- |
| Strongly disagree | Disagree | Neutral | Agree | Strongly Agree |

1. I think that using a computer or mobile health application to assist with maintaining or improving the health condition of my patients fits well within my clinical practice.

| 1 | 2 | 3 | 4 | 5 |
| --- | --- | --- | --- | --- |
| Strongly disagree | Disagree | Neutral | Agree | Strongly Agree |

1. It is easy for me to use a computer or mobile application to assist with maintaining or improving the health condition of my patients.

| 1 | 2 | 3 | 4 | 5 |
| --- | --- | --- | --- | --- |
| Strongly disagree | Disagree | Neutral | Agree | Strongly Agree |
